# Post-Vaccination Surveillance of Invasive Pneumococcal Disease in Ghana

**DOI:** 10.3390/diseases14050162

**Published:** 2026-05-07

**Authors:** Fleischer C. N. Kotey, Reuben E. Arhin, Nicholas T. K. D. Dayie, Emmanuel O. Ampah, Abass Abdul-Karim, Deric A. Baah, Ruth M. Afful, Georgina Tetteh-Ocloo, Roland T. Kom-Zuta, Francis K. M. Tetteh, Mary-Magdalene Osei, Yvonne N. A. Brew, Mame Y. Nyarko, Karikari Asafo-Adjei, Patience B. Tetteh-Quarcoo, Edem M. A. Tette, Eric S. Donkor

**Affiliations:** 1 Department of Medical Microbiology, University of Ghana Medical School, Korle Bu, Accra P.O. Box KB 4236, Ghana; fleischarles@gmail.com (F.C.N.K.); rearhin@atu.edu.gh (R.E.A.); ntkddayie@ug.edu.gh (N.T.K.D.D.); mishidaa1@gmail.com (E.O.A.); debaah1990@gmail.com (D.A.B.); ruthmamleafful@gmail.com (R.M.A.); ginatdasi@yahoo.com (G.T.-O.); rzuta2002@gmail.com (R.T.K.-Z.); tettehmorgan@gmail.com (F.K.M.T.); mmosei@ug.edu.gh (M.-M.O.); pbtetteh-quarcoo@ug.edu.gh (P.B.T.-Q.); 2 Department of Science Laboratory Technology, Accra Technical University, Accra P.O. Box GP 561, Ghana; 3 Greater Accra Regional Hospital, Accra P.O. Box 473, Ghana; yvonne.brew@ghs.gov.gh; 4 Zonal Public Health and Reference Laboratory, Tamale P.O. Box TL 99, Ghana; abdul-karim.abass@ghs.gov.gh; 5 Korle Bu Teaching Hospital, Korle Bu, Accra P.O. Box KB 77, Ghana; 6 Eastern Regional Hospital, Koforidua P.O. Box KF 175, Ghana; 7 Pathology Division, 37 Military Hospital, Neghelli Barracks, Accra P.O. Box 121, Ghana; 8 Princess Marie Louise Children’s Hospital, Accra P.O. Box GP 122, Ghana; mameyaanyarko@gmail.com; 9 Ho Regional Hospital, Ho P.O. Box MA 374, Ghana; kk.asafoadjei@gmail.com; 10 Department of Community Health, University of Ghana Medical School, Korle Bu, Accra P.O. Box KB 4236, Ghana; ematette@ug.edu.gh

**Keywords:** *Streptococcus pneumoniae*, invasive pneumococcal disease, serotype distribution, antimicrobial resistance, Ghana, post-vaccination epidemiology

## Abstract

Background: *Streptococcus pneumoniae*, also referred to as pneumococcus, is of immense public health significance. In particular, it causes severe invasive diseases among children. This has led to the recommendation of anti-pneumococcal prophylaxis, including the administration of penicillin and pneumococcal conjugate vaccines (PCVs), which have become available in about 90% of the countries in sub-Saharan Africa. Nonetheless, breakthrough disease still occurs. Also, PCVs can cause a shift in the distribution of pneumococcal serotypes, usually towards non-vaccine types. However, in many sub-Saharan African countries where PCVs have been introduced, there are hardly any comprehensive post-vaccination surveillance data on pneumococcus. Aim: To describe the post-vaccination epidemiology of invasive pneumococcal disease (IPD) in Ghana, including the prevalence, serotype distribution and antibiotic resistance. Methods: The study was cross-sectional and involved 14,597 patients recruited at the Korle Bu Teaching Hospital, Greater Accra Regional Hospital, Princess Marie Louise Children’s Hospital, Ho Regional Hospital, Eastern Regional Hospital, and Zonal Public Health and Reference Laboratory, Tamale. Specimens of cerebrospinal fluid (obtained by lumbar puncture) and blood were collected routinely from meningitis patients, while blood specimens were taken from pneumonia patients. These were cultured for *S. pneumoniae* following standard microbiological methods and subjected to antimicrobial susceptibility testing. The isolates were serotyped by the pneumotest latex agglutination kit, and the results confirmed by Quellung reaction, using serotype-specific antisera. Results: The overall prevalence of IPD was 0.66% (*n* = 97), varying across syndromes: bloodstream infections (0.53%, *n* = 38), meningitis (2.45%, *n* = 43), and pneumonia (0.28%, *n* = 16). The majority of the cases (56.70%, *n* = 55) occurred in the 11–20-year-old group. Ten pneumococcal serotypes were identified, with Serotype 1 being predominant (58.76%), followed by Serotypes 23B (11.34%), 33F (9.28%), and 12F (8.24%). Vaccine serotypes accounted for 81.44% of the isolates, while 18.56% were non-vaccine serotypes (23A, 23B, and 38). Antimicrobial resistance was highest against sulphamethoxazole-trimethoprim (52%), ampicillin (51%), and penicillin (46%). No resistance was observed against ciprofloxacin, levofloxacin, and vancomycin. The multidrug resistance proportion was 42.3% (*n* = 41). Conclusions: Even in the post-vaccination era, vaccine-type IPD remains a significant public health issue in Ghana. The observed serotype distribution and antimicrobial resistance patterns warrant sustained surveillance, more adaptive vaccination policies, and rigorous antibiotic stewardship to effectively mitigate IPD burden.

## 1. Introduction

*Streptococcus pneumoniae*, also referred to as pneumococcus, is a Gram-positive, lancet-shaped diplococcal pathogen and is of immense public health significance [1,2,3]. An important characteristic of the pathogen is its possession of a polysaccharide capsule, which defines about 100 capsular types and is the basis of current pneumococcal vaccines [4,5,6,7]. Though part of the normal bacterial flora of the upper respiratory tract [8,9,10,11,12,13], *S. pneumoniae* can spread and establish at various other anatomical sites and cause invasive and noninvasive diseases, including pneumonia, meningitis, septicaemia, sinusitis and acute otitis media. Invasive pneumococcal disease (IPD) is deadly, and it is usually caused by only a handful of *S. pneumoniae* serotypes, which seem to differ geographically [14,15]. Populations most at risk of IPD include children under 5 years, older people, individuals with heart disease, sickle cell disease or other kinds of splenic dysfunction, as well as immunodeficiency [3,16,17,18,19,20]. Each year, across the world, approximately 14.5 million severe pneumococcal disease episodes occur among children under five years old, about 500,000 cases of which are fatal, mostly in low- and middle-income countries [21]. The pneumococcal public health burden is further accentuated by increasing antimicrobial resistance (AMR) against essential antibiotics, especially penicillin, cephalosporins, and macrolides [22,23,24,25]. The high occurrence of pneumococcal AMR genes, coupled with the potential horizontal spread of these genes, has facilitated the dissemination of multidrug-resistant strains of the pathogen globally [22,23,24,25]. According to estimates, up to 40% of *S. pneumoniae* display multidrug-resistant (MDR) phenotypes, which is geographically heterogeneous [26]. In Ghana, the prevalence of multidrug resistance in *S. pneumoniae* has been reported to be as high as 87% [27].

Vaccination with pneumococcal conjugate vaccines (PCVs) is considered a major means of mitigating the burden of pneumococcal disease, especially among risk groups [28,29,30,31]. In sub-Saharan Africa, PCVs have so far been introduced in about 90% of the countries, though coverage levels are significantly disparate across countries [32,33]. Global epidemiological data show that following the inception of PCV use, there has been a drastic nosedive in IPD cases among vaccinated children in many settings, coupled with a herd immunity effect in other age groups [34,35,36]. For instance, the routine introduction of PCV13 over a 10-year period markedly reduced pneumonia and IPD incidence in rural Gambia; specifically, IPD incidence declined by 80% and 69%, respectively, among 2–59 months and 5–14 years old children [37]. Although PCVs have been instrumental in IPD burden reduction, they are hardly a panacea for pneumococcal syndromes, given that they cover 10–20 of the about 100 currently known serotypes [38,39,40]. A systematic review showed that 65–87% of pneumococcal serotypes that caused diseases in West Africa prior to the introduction of PCVs were covered by PCV13 [41]. In Ghana, a carriage study that serotyped the largest number of pneumococcal isolates prior to the introduction of the 13-valent pneumococcal conjugate vaccine (PCV13) reported a serotype coverage of 50% [42]. Moreover, following introduction of PCVs, there have been pneumococcal evolutionary responses that threaten public health benefits of the vaccines. Recombination at the capsular polysaccharide locus of the pneumococcus can result in capsular switching and vaccine-escape strains [43,44,45,46]. Evidence from several countries indicates that non-vaccine serotypes of the pneumococcus have been increasing in disease and carriage in the era of PCVs [47,48,49,50,51,52,53]. These observations highlight the need for post-vaccination surveillance of *S. pneumoniae*, particularly in areas where the pneumococcal disease burden is greatest, such as sub-Saharan African countries. Such surveillance data are crucial for informing pneumococcal vaccination policies, including the serotypes to include in new vaccine formulations. The aim of this study was to describe the post-vaccination epidemiology of *S. pneumoniae* invasive disease in Ghana, including the prevalence, serotype distribution and antibiotic resistance.

## 2. Materials and Methods

### 2.1. Study Site and Design

The study was carried out in Ghana, located in West Africa. Ghana has a population of 30 million people [54], and is considered a lower-middle-income country with a Gross National Income of $1098 [55]. The life expectancy in Ghana is about 65 years, and the literacy rate is 71.5% [54,55]. Within the country are over 100 hospitals, in many of which a national health insurance scheme has been operational since 2004 [54,55]. Ghana initiated routine PCV13 immunisation in 2012 and the vaccine is administered through the Expanded Programme of Immunisation (EPI) and hospital-based programmes. In accordance with guidelines of the World Health Organisation, the vaccine is administered to infants on their 6th, 10th, and 14th weeks of life [4]. On average, the coverage of PCV13 vaccination in Ghana is about 99% (http://www.view-hub.org). Apart from PCV13, no other pneumococcal conjugate vaccines have been previously used in Ghana.

This was a cross-sectional study involving IPD surveillance at seven sites in Ghana, including the Korle Bu Teaching Hospital (KBTH), Greater Accra Regional Hospital (GARH), Princess Marie Louise Children’s Hospital (PML), 37 Military Teaching Hospital (37MH), Ho Regional Hospital (HRH), Eastern Regional Hospital (ERH), and Zonal Public Health and Reference Laboratory, Tamale (ZPHRLT). The selection of these sites was based on their geographical locations, which adequately cover the country, as well as the high patient turnout and their capacity for isolation of *Streptococcus pneumoniae* from clinical specimens. KBTH, GARH, PML, HRH, and ERH are located at the extreme south, while ZPHRLT is at the northern part of the country.

### 2.2. Patient Recruitment and Specimen Collection

Surveillance for IPD was set up at the study sites for 36 months (1 January 2020 to 31 December 2022). All patients diagnosed with pneumonia, meningitis, septicaemia, and any other invasive infections at each of the sites were recruited into the study. The case definitions recommended by the World Health Organisation were used in the diagnosis of these infections. Patients were excluded if informed consent could not be obtained or if specimens were deemed unsuitable for analysis due to contamination or inadequate volume. The patients’ clinical information was collected via a review of their folders. Specimens of CSF (obtained by lumbar puncture) and blood were collected routinely from meningitis patients, and blood from patients with bloodstream infections. Blood and sputum specimens were taken from pneumonia patients; in addition, children with pneumonia also had chest X-ray performed. Clinical specimens were collected at participating sites and transported to the laboratory under appropriate temperature-controlled conditions. Samples were processed promptly upon arrival to preserve bacterial viability. When immediate processing was not possible, specimens were stored under conditions consistent with standard microbiological guidelines.

### 2.3. Laboratory Investigations

Initial specimen analyses to isolate *S. pneumoniae* and perform antimicrobial susceptibility testing (AST) were done at the participating sites. CSF specimens were cultured on 5% sheep blood agar and chocolate agar (Oxoid, Basingstoke, UK). Blood samples were processed using an automated culture system (BACTEC 9060 [Becton, Dickinson and Company [BD], Sparks, MD, USA]), and cultures with a positive signal were sub-cultured by standard methods on 5% sheep blood agar and chocolate agar (Oxoid, Basingstoke, UK). Sputum specimens were cultured on 5% sheep blood agar and chocolate agar (Oxoid, Basingstoke, UK) supplemented with gentamicin. *S. pneumoniae* isolates were identified initially by colony morphology and Gram-stain and confirmed by bile solubility and optochin inhibition [56,57]. Antimicrobial susceptibility testing (AST) was performed using both the Kirby–Bauer disc diffusion and E-test methods. Specifically, Kirby–Bauer was used for tetracycline (30 μg), erythromycin (15 μg), trimethoprim–sulfamethoxazole (1.25/23.75 μg), and cefotaxime (30 μg) (Oxoid, Basingstoke, UK), while E-test was applied for penicillin, ampicillin, amoxicillin–clavulanate, amoxicillin, ciprofloxacin, levofloxacin, and vancomycin (bioMérieux, Durham, NC, USA). Each antibiotic was tested against all 97 confirmed *S. pneumoniae* isolates, and *S. pneumoniae* ATCC 49619 (Manassas, VA, USA) was used as quality control. Inhibition zone sizes from the Kirby–Bauer tests and minimum inhibitory concentrations from the E-tests were interpreted based on the breakpoint criteria of the Clinical and Laboratory Standards Institute (CLSI) [58].

The isolates were confirmed as pneumococci by the MALDI TOF (BRUKER Daltonics GmbH & Co. KG, Bremen, Germany) at the Department of Medical Microbiology, University of Ghana Medical School, Accra. They were serotyped by the pneumotest latex agglutination kit (SSI Diagnostica, Hillerød, Denmark), and the results confirmed by the Quellung reaction, using serotype-specific antisera (SSI Diagnostica). Serotyping of the isolates was done at the Zonal Public Health and Reference Laboratory, Tamale, in conjunction with the Department of Medical Microbiology, University of Ghana Medical School.

### 2.4. Data Analysis

The data collected were entered into MS Excel and exported to STATA, Version 14.2 (StataCorp, College Station, TX, USA) for analyses. The strategies taken to analyse the data involved descriptive statistics, including means and standard deviations, frequencies, proportions, and 95% confidence intervals, as appropriate. Specific analyses were carried out to determine the prevalence of *S. pneumoniae* for bloodstream infections, meningitis, pneumonia, and IPD. Associations between age group and IPD status were assessed using the Chi-square test of independence. Statistical significance was defined as *p* < 0.05. Serotypes and antibiotic resistance were evaluated in terms of frequencies and proportions. Missing data were minimal and were excluded from analyses on a case-by-case basis. No multivariable adjustments or formal trend analyses were performed.

### 2.5. Ethical Considerations

Ethical approval was obtained from the Ethical and Protocol Review Committee of the College of Health Sciences, University of Ghana (Protocol ID: CHS-Et/M.5—P4.5/2021-2022). Informed consent was obtained from the study participants and their parents (in the case of children).

## 3. Results

### 3.1. Demographic Features of the Patients Recruited in the Study

Over the study period, 14,597 patients were recruited from the various study hospitals, including KBTH (3742), GARH (2180), TRL (895), 37MH (181), PML (5715), ERH (938), and HRH (946) (Table 1). The age range of the patients was 0.20 to 78 years, with a mean of 22.51 ± 16.81 years; 13.78% (*n* = 2012) of the patients were children aged 5 years or younger, while 7513 (54.47%) were adults aged 18 years and older. A proportion of 49.70% (*n* = 7259) of the recruited patients were males, while 50.30% (*n* = 7338) were females.

### 3.2. Clinical Features of the Patients Recruited in the Study

Of the 14,597 IPD-suspected patients who participated in the study, only 23.0% (*n* = 3354), all of whom were aged 10 years or younger, had received PCV13 vaccination. A smaller proportion (12.03%; *n* = 1756; 95% CI = 0.115–0.126) were diagnosed with meningitis, whereas the majority were diagnosed with bloodstream infections (48.92%; *n* = 7142; 95% CI = 0.481–0.497) or pneumonia (39.04%; *n* = 5699; 95% CI = 0.383–0.398). The age group with the highest proportion of meningitis cases was 21–30 years, accounting for 29.50% (*n* = 518; 95% CI = 0.274–0.316) of all meningitis cases. That age group also had the highest proportion of bloodstream infection cases 35.38% (*n* = 2527; 95% CI = 0.343–0.365) of all BSI cases, while regarding pneumonia, the 11 to 20 years age group recorded the highest number of cases (54.41%, *n* = 3101; 95% CI = 0.531–0.557). In terms of sex, females accounted for a slightly higher proportion of the meningitis (50.40% vs. 49.60%; 95% CI = 0.481–0.527), BSI (50.30% vs. 49.70%; 95% CI = 0.491–0.515), and pneumonia cases (50.20% vs. 49.80%; 95% CI = 0.489–0.515).

Overall, there was a general fluctuation in the number of bloodstream infections reported each month over the years, with some months showing higher counts than others (Supplementary Figure S1). Generally, though, the number of cases recorded during the dry seasons was lower than that recorded during the wet seasons. While fluctuations were observed in the number of meningitis cases as well, a peak in the number of cases was observed for June 2021 (402 cases), as well as in the wet season of 2021 (617 cases) (Supplementary Figure S2). Regarding the pneumonia cases, peaks in the number of cases were observed in December 2022 (1611 cases) and the dry season of 2022 (2458 cases), amidst general fluctuations during the study period (Supplementary Figure S3).

### 3.3. Prevalence of IPD, Causative Serotypes, and AMR

The frequencies of *S. pneumoniae* BSIs, meningitis, and pneumonia among the participants were 0.53% (*n* = 38; 95% CI = 0.39–0.73%), 2.45% (*n* = 43; 95% CI = 1.82–3.28%), and 0.28% (*n* = 16; 95% CI = 0.17–0.46%), yielding an overall IPD prevalence of 0.66% (*n* = 97; 95% CI = 0.54–0.81%). The distribution of IPD cases differed significantly across age groups (χ^2^ = 74.04, df = 8, *p* < 0.001) (Table 2). The highest proportion of cases was observed among adolescents and young adults aged 11–20 years (Table 2).

Overall, ten pneumococcal serotypes were detected among the confirmed IPD cases, namely Serotypes 1, 12F, 33F, 14, 20, 23A, 23B, 10A, 38, and 8, with Serotype 1 being the modal serotype, and the bulk of the cases (*n* = 55, 56.70%) were seen in the 11 to 20 years age group (Table 2 and Table 3).

Moreover, the vaccine serotypes were the predominant serotypes observed across all the age groups, with only three of the serotypes—Serotypes 23A, 23B, and 38—unaccounted for in the existing pneumococcal vaccines (Table 3).

**Figure 1:**
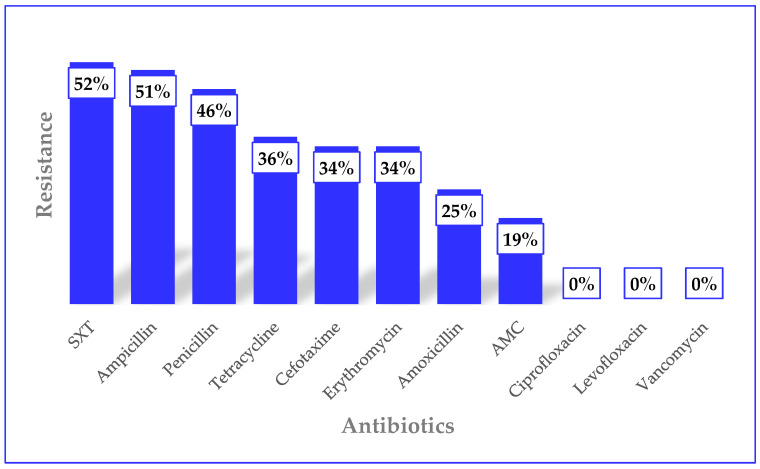
AMR patterns of the pneumococci. In the figure, SXT = Sulphamethoxazole–trimethoprim, and AMC = Amoxicillin–clavulanate.

With regard to AMR, resistance rates, in decreasing order, were observed for sulphamethoxazole–trimethoprim (SXT) (52%, *n* = 50), ampicillin (51%, *n* = 49), penicillin (46%, *n* = 45), tetracycline (36%, *n* = 35), cefotaxime and erythromycin (34% each, *n* = 33), amoxicillin (25%, *n* = 24), amoxicillin–clavulanate (19%, *n* = 18), and ciprofloxacin, levofloxacin, and vancomycin (0% each, *n* = 0) (Figure 1). The frequency of multidrug resistance (defined as non-susceptibility to three or more classes of antibiotics) was 42.3% (*n* = 41).

Among the 97 IPD cases, 62% (*n* = 60) had documented prior antibiotic use. The most common antibiotics previously administered included penicillin, ampicillin, and trimethoprim–sulfamethoxazole.

## 4. Discussion

The purpose of this study was to investigate the post-vaccination epidemiology of invasive pneumococcal disease (IPD) in Ghana, focusing on disease prevalence, antimicrobial resistance patterns, and circulating pneumococcal serotypes. This study addresses important knowledge gaps, as the limited insights into the epidemiology of the pneumococcus in the country have mainly emanated from carriage studies [27,42,57,59,60,61,62,63,64,65,66].

The seasonal variations observed, with higher numbers of cases during wet seasons for bloodstream infections and meningitis, align with previous reports from the Meningitis Belt of Africa, where Ghana is partially located. While these seasonal variations pertain to all the recruited patients, Leimkugel et al. [67] and Kwambana-Adams et al. [61] have reported similar seasonal patterns in pneumococcal disease in Northern Ghana, attributing this to climatic factors that affect transmission dynamics.

The overall prevalence of IPD in this study (0.66%) was low, with marked heterogeneity across the three syndromes—bloodstream infections (0.53%), meningitis (2.45%), and pneumonia (0.28%). Notably, only one IPD case was detected among the 1–5-year-old group and none in infants. This finding likely reflects the impact of Ghana’s routine infant PCV13 immunisation programme, which has achieved high coverage and is expected to confer direct protection in this age group [42,57,68].

In contrast, a higher burden of IPD was observed among individuals aged 11–20 years. This differs from the typical U-shaped age distribution of IPD in many settings, where the highest burden is often observed among young children and the elderly [69]. It may reflect a cohort effect associated with the introduction of PCV13 in 2012. Individuals aged 10 years or younger were more likely to have been vaccinated, as reflected in our dataset, whereas those aged 11–20 years were largely not included in a catch-up vaccination programme. This likely results in lower vaccine-derived protection in this cohort and may partly explain the observed disease burden. However, population-level vaccination coverage data for older children and adolescents were not available, and this interpretation should be made with caution.

The temporal distribution of IPD cases observed in this study is likely influenced by a combination of factors rather than a single determinant. These include changes in circulating pneumococcal serotypes following the introduction of PCV13, natural secular trends in pneumococcal transmission, capsular switching, heterogeneous vaccine coverage across regions and age cohorts, and host-related factors such as age-dependent exposure patterns and immune responses [45,46,70,71,72,73,74,75,76]. Similar multifactorial influences on pneumococcal disease dynamics have been described in other post-vaccination settings, particularly within the African Meningitis Belt.

Although an increased burden of IPD was observed among adolescents and young adults, this study was not designed to directly assess the durability of PCV13-induced immunity. As such, waning vaccine-induced protection cannot be inferred from these findings. Rather, the observed age shift likely reflects a complex interplay between historical vaccination eligibility, incomplete catch-up coverage in older cohorts, and ongoing transmission of highly invasive serotypes such as Serotype 1 [6,70,71,72]. Nonetheless, this epidemiological shift underscores the importance of sustained post-vaccination surveillance to detect temporal changes in disease burden and serotype distribution, particularly in low- and middle-income settings where transmission dynamics, population structure, and vaccine impact may differ from those observed in high-income countries.

Serotype 1, one of the vaccine serotypes, was the predominant cause of IPD in this study, consistent with previous reports from Ghana and other countries within the African Meningitis Belt [61,67,71,77]. Although non-vaccine pneumococci are known to be the main drivers of the pneumococcal disease burden after pneumococcal vaccine introduction, there were few non-vaccine serotype IPD cases in this study, highlighting an ongoing potential for serotype replacement [78,79]. Unlike many pneumococcal serotypes, Serotype 1 is characterised by high invasive potential but relatively low nasopharyngeal carriage, which may limit the indirect herd protection conferred by infant vaccination [10,27,50]. Its persistence following PCV13 introduction has been widely reported and does not necessarily indicate vaccine failure but rather reflects the distinct transmission and pathogenic dynamics of this serotype [10,27,50,57,59,62,63]. These characteristics underscore the importance of continued monitoring of Serotype 1 in post-vaccination settings. Apart from post-vaccine carriage studies, the vaccine immune responsiveness to PCV13 in Ghanaian recipients has not been studied; such investigations are warranted in future studies to ascertain how effective PCV13 has been locally. Voysey et al. [80] and Swarthout et al. [81] have reported that in low- and middle-income countries, correlates of protection in pneumococcal vaccine recipients are about twice as high as in high-income countries. Therefore, there is a need to ascertain the vaccine immune responsiveness to Serotypes 1 and 14 in Ghanaian populations especially, as these serotypes are captured in PCV13, which has been in use in Ghana for over a decade.

Another focus of this study was to evaluate the AMR patterns of the pneumococcus involved in the IPD cases. The penicillin AMR rate in this study (46%) suggests that penicillin may not be a suitable option for treatment of the majority of IPD cases reported in this study. Von Specht et al. [82] have identified that the expansion of penicillin-resistant pneumococcal clones differs between regions and is promoted by usage of antibiotics, conjugate vaccines, and population density. Similarly to the phenomenon with penicillin, a high resistance rate (51%) was observed against ampicillin, which is a derivative of penicillin, suggesting limited clinical utility for empirical treatment of IPD in the study setting, particularly when compared with alternative agents. The CLSI [58] recommends the use of maximum doses of cefotaxime for treatment of both pneumococcal meningitis and non-meningitis cases. In this study, cefotaxime resistance was moderate (34%) and may, thus, be a better option.

Resistance to erythromycin in this study was found to be relatively low (34%). Higher proportions of 62.3% have been reported for invasive isolates in Tunisia [83], whereas a resistance of 17.9% has been reported among post-vaccine era carriage isolates in Morocco. In a multicentre evaluation of pneumococcal antibiotic resistance in India, a decrease in the rate of erythromycin-susceptible pneumococci from 71.43% to 16.22% has been previously reported [84], which suggest that erythromycin resistance is on the increase in some regions.

Generally, oral tetracyclines are commonly considered when treating adult lower respiratory tract infection in outpatients, including those presenting with community-acquired pneumonia (CAP) following previous guideline-concordant treatment options for oral CAP [85]. Tetracycline resistance was moderate (36%), and this is reflective of other higher rates in countries such as China, where a rate as high as 98.3% has been reported for invasive and noninvasive isolates [86].

Amoxicillin is a first-line oral antibiotic for treating bacteria-related lower respiratory tract infections [87]. The amoxicillin–clavulanate combination is indicated for the treatment of pneumococcal non-meningitis cases according to the CLSI guidelines [58]. The in vitro success rates show that pneumococcal resistance to this treatment option was low and, therefore, supports its use over the single amoxicillin therapy.

Trimethoprim–sulfamethoxazole (cotrimoxazole) is recommended by the WHO as a first-line antimicrobial agent for treating respiratory tract infections [88]. A rate of 18.2% sterile site cotrimoxazole-non-susceptible pneumococci has been previously reported from the CDC’s Active Bacterial Core surveillance data [89]. This suggests that cotrimoxazole resistance was relatively high among invasive isolates in the study population. A study has identified that in Ghana, the existing guidelines on antibiotic usage, as prescribed in the Essential Medicines List (EML), Standard Treatment Guidelines (STGs), and the National Health Insurance Scheme Medicines List (NHISML), are mostly not adhered to [90], and hence, this may account for the high resistance observed against some antibiotics, such as cotrimoxazole.

Both levofloxacin and vancomycin showed considerable promise for effective management of invasive pneumococcal infections. However, vancomycin is considered in combination with therapy with an aminoglycoside such as gentamicin [91] or with ceftriaxone for treatment of penicillin-non-susceptible pneumococci [92].

Multidrug resistance, which is currently on the rise among the pneumococci and most bacterial pathogens, was high among the pneumococci in this study [47,93,94]. This observation, along with the AMR trend observed in connection with the other antibiotics tested in this study, lends further support to calls for optimisation of antimicrobial stewardship programmes. It is noted, though, that the selection of antibiotics tested in this study reflects those routinely used or available in the study setting for the management of suspected invasive pneumococcal infections. However, this represents a limitation, as resistance to some clinically relevant agents was not evaluated. In particular, pleuromutilins such as lefamulin, which have demonstrated efficacy against highly penicillin-resistant pneumococci in adults, were not assessed. While ceftriaxone was not included, cefotaxime—an antibiotic with a similar spectrum and clinical use—was tested. The absence of aminoglycosides such as gentamicin is of limited relevance, given their poor intrinsic activity against pneumococcus and their restricted role as an adjunctive therapy in severe cases. In addition, the observational design of the study, as well as the uneven geographic distribution of the study sites, may limit the generalisability of the findings. Also, the reliance on culture-based methods may have led to an underestimation of pneumococcal detection, as *S. pneumoniae* is prone to autolysis. Moreover, variations in case ascertainment and healthcare-seeking behaviour across regions could have influenced the reported prevalence rates. That notwithstanding, the study has significant strengths—it is one of the few robust studies examining post-vaccination pneumococcal epidemiology in Ghana, providing much-needed insights on pneumococcal serotype distribution and AMR patterns. Also, its multicentre approach, which involved seven healthcare facilities across different regions, enhances the representativeness of the findings. In addition, the inclusion of both paediatric and adult populations allows for age-specific analyses that could inform targeted interventions.

## 5. Conclusions

There was a relatively low overall IPD prevalence in post-vaccination Ghana, but with a notable shift in the age distribution of cases toward adolescents and young adults. This is likely attributable to the absence of pneumococcal vaccination in these age cohorts, as Ghana’s PCV13 programme primarily targets infants. The continued predominance of vaccine serotypes, particularly Serotype 1, suggests the need for expanded vaccination coverage beyond early childhood. Moreover, the observed high rates of AMR to commonly used antibiotics underscore the importance of antibiotic stewardship and informed empirical treatment guidelines for pneumococcal infections in the country.

## Figures and Tables

**Table 1:** Demographic features of the recruited patients.

Feature		Number	%
Age groups (years)	<1	53	0.36
	1–5	1959	13.42
	6–10	1342	9.19
	11–20	4175	28.60
	21–30	3433	23.52
	31–40	1097	7.52
	41–50	1155	7.91
	51–60	1087	7.45
	>60	296	2.03
Sex	Male	7259	49.70
	Female	7338	50.30
Region	Greater Accra	11,818	80.96
	Eastern	938	6.43
	Volta	946	6.48
	Northern	895	6.13
Study site	GARH	2180	14.93
	KBTH	3742	25.64
	37 MH	181	1.24
	PML	5715	39.15
	HRH	946	6.48
	ERH	938	6.43
	ZPHRLT	895	6.13

Mean age (bloodstream infection cases = 26.46 ± 18.57 years; meningitis cases = 22.70 ± 16.05 years; pneumonia cases = 17.49 ± 12.96 years; overall = 22.51 ± 16.81 years); GARH = Greater Accra Regional Hospital; KBTH = Korle Bu Teaching Hospital; 37 MH = 37 Military Teaching Hospital; PML = Princess Marie Louise Children’s Hospital; HRH = Ho Regional Hospital; ERH = Eastern Regional Hospital; ZPHRLT = Zonal Public Health and Reference Laboratory, Tamale.

**Table 2:** Distribution of the vaccine- and non-vaccine pneumococcal serotypes by age group and gender.

Features	Frequency of Pneumococci (*n* = 97)	
	Vaccine Serotypes *n* (%)	Non-Vaccine Serotypes *n* (%)
Age groups **		
<1 year (*n* = 0)	0 (0.00%)	0 (0.00%)
1 to 5 years (*n* = 1)	1 (100.00%)	0 (0.00%)
6 to 10 years (*n* = 6)	4 (66.67%)	2 (33.33%)
11 to 20 years (*n* = 55)	48 (87.27%)	7 (12.73%)
21 to 30 years (*n* = 5)	5 (100.00%)	0 (0.00%)
31 to 40 years (*n* = 16)	9 (56.25%)	7 (43.75%)
41 to 50 years (*n* = 8)	6 (75.00%)	2 (25.00%)
51 to 60 years (*n* = 1)	1 (100.00%)	0 (0.00%)
>60 years (*n* = 5)	5 (100.00%)	0 (0.00%)
Gender **		
Male (*n* = 55)	48 (87.27%)	7 (12.73%)
Female (*n* = 42)	39 (92.86%)	3 (7.14%)

** The “*n*” value of each row was used as the denominator when computing the proportion within each row; number of vaccine serotypes = 79; number of non-vaccine serotypes = 18. Differences across age groups were statistically significant (χ^2^ = 74.04, df = 8, *p* < 0.001).

**Table 3:** General distribution of the pneumococcal serotypes.

Serotype	No.	%	95% CI (%)	Vaccines Within Which the Serotypes Are Captured
1	57	58.76	48.82–68.19	PCVs 10, 13, 15, 20, and PPV 23
8	1	1.03	0.002–0.056	PCV 20, PPV 23
10A	1	1.03	0.002–0.056	PCV 20, PPV 23
12F	8	8.24	0.042–0.155	PCV 20, PPV 23
14	1	1.03	0.002–0.056	PCVs 10, 13, 15, 20, and PPV 23
20	2	2.06	0.006–0.071	PPV 23
23A	3	3.09	0.011–0.086	–
23B	11	11.34	0.065–0.192	–
33F	9	9.28	0.050–0.167	PCVs 15 and 20, PPV 23
38	4	4.12	0.016–0.101	–

## Data Availability

The data underlying this study are available upon reasonable request via the corresponding author at esampane-donkor@ug.edu.gh.

## Supplementary Materials

The following supporting information can be downloaded at: https://www.mdpi.com/article/10.3390/diseases14050162/s1, Figure S1: Monthly and seasonal pattern of BSI cases across the study period; Figure S2: Monthly and seasonal patterns of meningitis cases across the study period; Figure S3: Monthly and seasonal patterns of pneumonia cases across the study period.

## Abbreviations

The following abbreviations are used in this manuscript:

AMC	Amoxicillin–clavulanate
AMR	Antimicrobial resistance
AST	Antimicrobial susceptibility testing
BSI	Bloodstream infection
CAP	Community-acquired pneumonia
CDC	Centres for Disease Control and Prevention
CLSI	Clinical and Laboratory Standards Institute
CSF	Cerebrospinal fluid
EML	Essential Medicines List
ERH	Eastern Regional Hospital
GARH	Greater Accra Regional Hospital
HRH	Ho Regional Hospital
KBTH	Korle Bu Teaching Hospital
IPD	Invasive pneumococcal disease
MDR	Multidrug-resistant
NHISML	National Health Insurance Scheme Medicines List
PCV	Pneumococcal conjugate vaccine
PML	Princess Marie Louise Children’s Hospital
PPV	Pneumococcal polysaccharide vaccine
SXT	Sulphamethoxazole–trimethoprim
STGs	Standard Treatment Guidelines
WHO	World Health Organization
ZPHRLT	Zonal Public Health and Reference Laboratory, Tamale
