# Supplementary material for: Post-Vaccination Surveillance of Invasive Pneumococcal Disease in Ghana
Source: Diseases. 2026 May 7;14(5):162. doi: 10.3390/diseases14050162 (PMC13205543; doi:10.3390/diseases14050162)
Supplement: Supplementary file 1 [file diseases-14-00162-s001.zip › diseases-4172558-supplementary.pdf]

Supplementary Figures

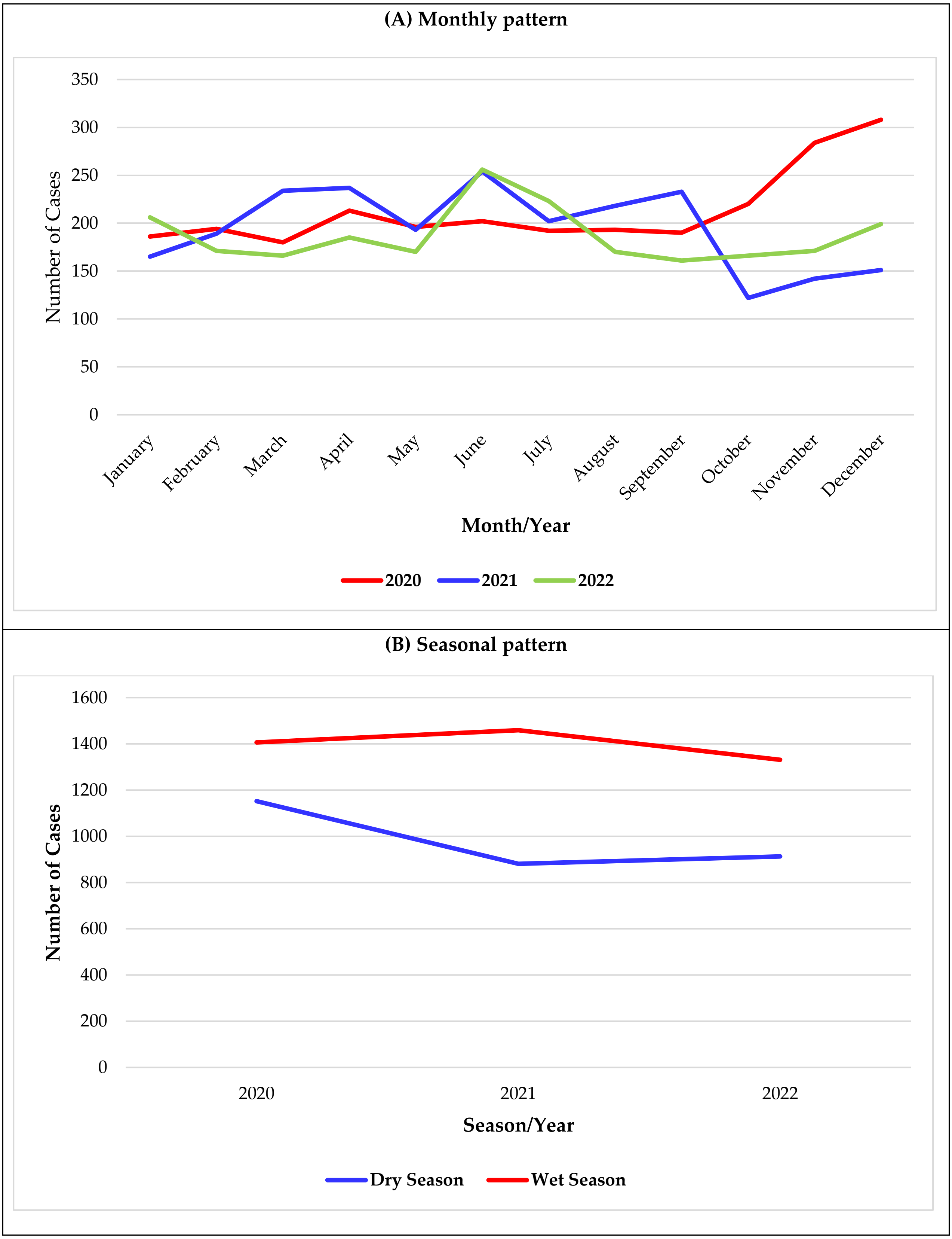

Supplementary Figure S1. Monthly and seasonal pattern of BSI cases across the study period.
Dry season = November to March; Wet Season = April to December

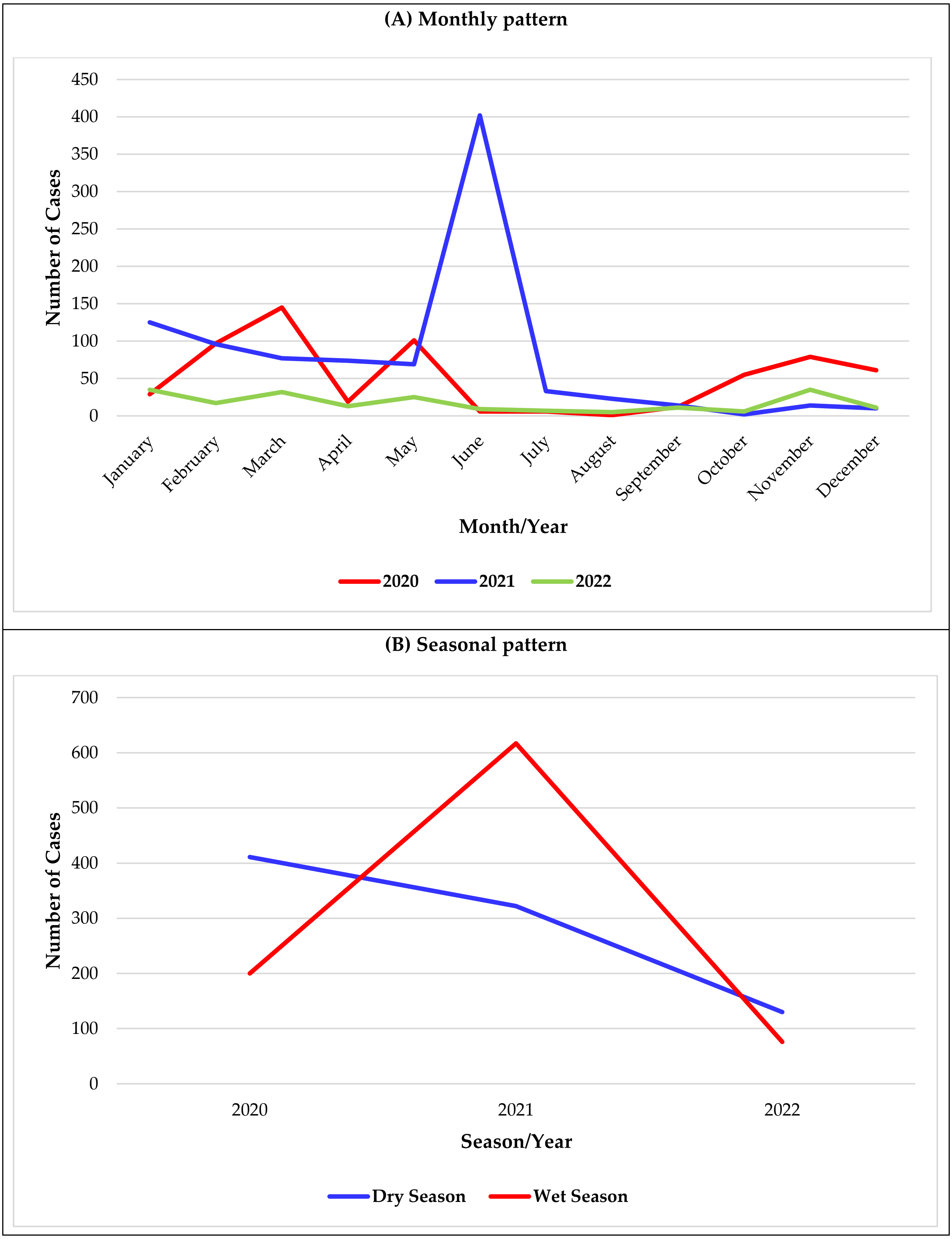

Supplementary Figure S2. Monthly and seasonal patterns of meningitis cases across the study period. Dry season = November to March; Wet Season = April to December

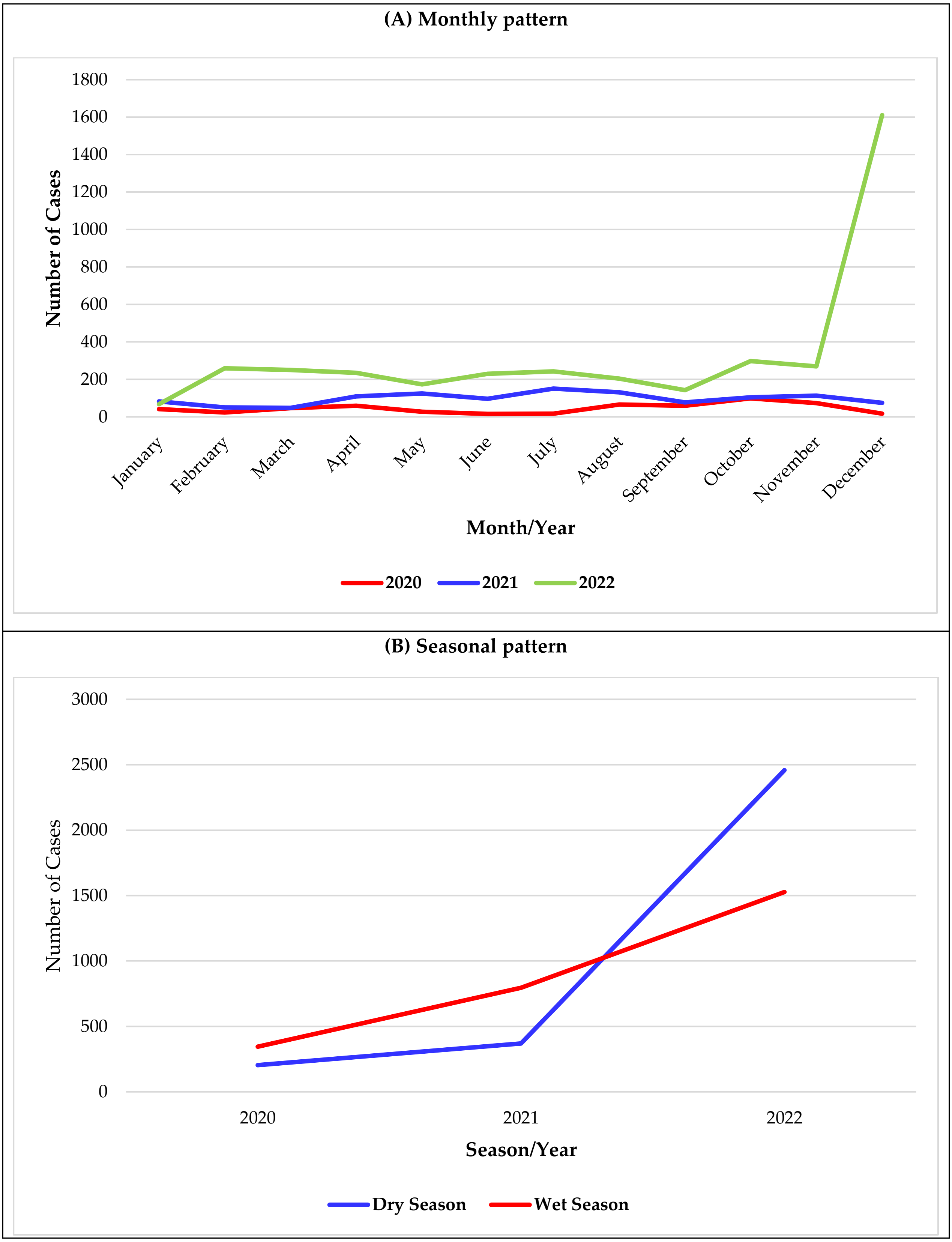

Supplementary Figure S3. Monthly and seasonal patterns of pneumonia cases across the study period. Dry season = November to March; Wet Season = April to December
